# CXCR4 expression on circulating pan-cytokeratin positive cells is associated with survival in patients with advanced non-small cell lung cancer

**DOI:** 10.1186/1471-2407-9-213

**Published:** 2009-06-29

**Authors:** Karen L Reckamp, Robert A Figlin, Marie D Burdick, Steven M Dubinett, Robert M Elashoff, Robert M Strieter

**Affiliations:** 1 Department of Medical Oncology & Therapeutics Research, City of Hope and Beckman Research Institute, Duarte, USA; 2 Department of Hematology & Hematopoeitic Transplantation Medicine, City of Hope and Beckman Research Institute, Duarte, USA; 3 Department of Medicine, University of Virginia School of Medicine, Charlottesville, USA; 4 Division of Pulmonary and Critical Care Medicine, David Geffen School of Medicine at UCLA, Los Angeles, USA; 5 Department of Pathology and Laboratory Medicine, David Geffen School of Medicine at UCLA, Los Angeles, USA; 6 Department of Biomathematics, David Geffen School of Medicine at UCLA, Los Angeles, USA

## Abstract

**Background:**

The CXC chemokine, CXCL12, and its receptor, CXCR4 promote metastases of a variety of solid tumors, including non-small cell lung cancer (NSCLC). The expression of CXCR4 on tumor cells may represent a critical biomarker for their propensity to metastasize. This study was performed to evaluate the hypothesis that co-expression of pan-cytokeratin and CXCR4 may be a prognostic marker for patients with advanced NSCLC.

**Methods:**

We evaluated CXCR4 levels on circulating pan-cytokeratin positive cells from patients with NSCLC. NSCLC tumor and metastases were also assessed for the presence of CXCR4.

**Results:**

Pan-cytokeratin positive cells were increased in the circulation of patients with NSCLC, as compared to normal control subjects. Patients with pan-cytokeratin +/CXCR4+ = 2,500 cells/ml had a significant improvement in median survival when compared with patients with pan-cytokeratin +/CXCR4+ >2,500 cells/ml (not achieved versus 14 weeks). CXCR4 expression was found on NSCLC tumors and at sites of tumor metastasis.

**Conclusion:**

This study suggests that CXCR4 may be a prognostic marker in NSCLC, and provides hypothesis-generating results, which may be important in determining metastatic potential. In future studies, we will prospectively evaluate the prognostic significance of pan-cytokeratin/CXCR4+ cells, and determine the mechanisms involved in the regulation of CXCR4 expression on tumor cells in a larger patient population.

## Background

Approximately 213,380 new cases of lung cancer will be diagnosed and 160,390 deaths will occur from lung cancer during 2007. Lung cancer is the leading cause of cancer death among both men and women in the United States [1]. Nearly 60% of those diagnosed with lung cancer die within one year of their diagnosis, and the five-year survival for all patients with lung cancer is only 16%. This statistic has not improved significantly in the past 10 years. Surgical resection offers the most promising chance for cure in patients who present with early-stage disease, although the majority of patients will develop recurrence despite complete surgical resection. This is likely secondary to undetected microscopic metastatic disease at the time of surgery, and recently adjuvant chemotherapy has been shown to improve survival in some early-stage patients [2-4]. Identification of targeted treatments for micrometastatic disease could block tumor cell migration and improve outcomes in this devastating disease.

Cytokeratins have been identified as potential markers for the detection of circulating cancer cells by RT-PCR, flow cytometry and RT-PCR [5-7]. Cytokeratin 19 has been described in the serum of patients with non-small cell lung cancer (NSCLC), and has been associated with tumor burden and response to therapy [7,8]. However, cytokeratins can be expressed in low levels in peripheral blood mononuclear cells, which limits the utility of this biomarker to accurately detect circulating micrometastatic disease.

Chemokines within the tumor microenvironment, and at sites of metastases can mediate invasion of tumor cells through mechanisms similar to those used in leukocyte stem cell trafficking [9]. Chemokines are a family of 8–11 kd proteins, subdivided on the basis of the position of the N-terminus cysteine residues. They are involved in leukocyte chemotaxis and activation, and have been associated with the regulation of angiogenesis and tumor cell invasion [10-12]. Chemokine receptors are seven transmembrane, G protein-coupled receptors, and are expressed on leukocytes, endothelial cells, stromal cells, epithelial cells and tumor cells [13,14]. Chemokine gradients within the tumor microenvironment and target organs can determine the metastatic potential of a tumor [15].

CXCR4 has been the major chemokine receptor expressed on cancer cells [16-20]. Its ligand, CXCL12 is essential for the homing of hematopoeitic stem cells during embryogenesis and adult life [21,22]. Müller and colleagues provided initial evidence linking CXCL12/CXCR4 biological axis to breast cancer metastasis to specific organs [16], which was confirmed in non-small lung cancer [20]. More recent studies have suggested that CXCR4 is expressed on various other cancer cells and its expression stimulated migration of cancer cells towards a CXCL12 gradient established in specific target organs for metastases [17-19]. The CXCR4/CXCL12 biological axis has been postulated to have an important in supporting cancer stem cells [23]. These findings suggest that CXCL12/CXCR4 may be a critical determinant for the metastatic potential of NSCLC, and supports the notion that the magnitude of pan-cytokeratin+ circulating cells that express CXCR4 will correlate with survival of patients with NSCLC. In this study we tested this hypothesis and found that the magnitude of circulating pan-cytokeratin+/CXCR4+ cells in patients with NSCLC was directly correlated with worse prognosis, and that these findings could be used as a foundation for future prospective biomarker studies. Furthermore, these results support the contention that further understanding the molecular mechanisms that are involved in the regulation of CXCR4 expression on tumor cells could lead to potential targets to modify the expression of CXCR4 on NSCLC cells and impact on metastases.

## Methods

### Study Design

Sixteen subjects with NSCLC and 10 normal healthy donors seen at UCLA Medical Center between October 2004 and March 2006 were enrolled. The UCLA institutional review board approved this study protocol, and all patients provided written informed consent. Peripheral blood was collected from patients prior to initiating systemic therapy.

### FACS analysis

Each specimen was processed to isolate buffy coat leukocyte populations for intracellular staining and FACS analysis. Briefly, buffy coat was isolated, red blood cells lysed, and the cells stained with APC anti-CXCR4 (R&D systems, Minneapolis, MN) or isotype control. Cells were then permeabilized with a BD Cytofix/Cytoperm kit (BD Biosciences) and stained with PE anti-Pan-cytokeratin (BD Biosciences). Samples were analyzed on a FACSCalibur instrument using Cellquest 3.2.1f1 software (BD Biosciences).

### Immunohistochemistry

Paraffin-embedded tissue microarrays (Lung: cancer-metastasis-normal) were purchased from Imgenex Corp. (San Diego, CA). The arrays were processed for immunohistochemical localization of CXCR4, using monoclonal anti-human CXCR4 antibodies or IgG2b antibodies for negative control (R&D Systems) as using Vector Elite Kit (Vector Laboratories, Burlingame, California, USA) as described previously [24]. Briefly, the tissue array was incubated with a 1:1 mixture of 3% hydrogen peroxide in methanol. The array was then exposed to Power Block (BioGenex Laboratories, San Ramon, California, USA) for 30 minutes and, after washing in PBS, stained with CXCR4 antibodies or an isotype control antibody for 30 minutes at room temperature. After incubation with the primary Ab, the array was then exposed to a biotinylated anti-mouse secondary Ab for 30 minutes. The array was washed in PBS and avidin-binding complex (Vector Laboratories) reagent was added, and the array incubated again for 30 minutes. The array was then exposed to the chromogen 3,3'-diaminobenzidine tetrahydrochloride (Vector Laboratories), which turns the positively staining cells brown. Finally, the array was counterstained with hematoxylin and cover-slipped.

### Statistical Analysis

Data were analyzed on a Dell PC computer using SAS version 9.2 XP_PRO platform. Non parametric Kruskal-Wallis test was used to detect the group differences because of small sample size. A survival curve was estimated according to the Kaplan-Meier method and groups compared with the log rank test. A p-value = 0.05 was considered significant.

## Results

### CXCR4 is Markedly Expressed on NSCLC Primary Tumor and in Cells of Metastatic Lesions from NSCLC tissue microarrays

**Figure 1:**
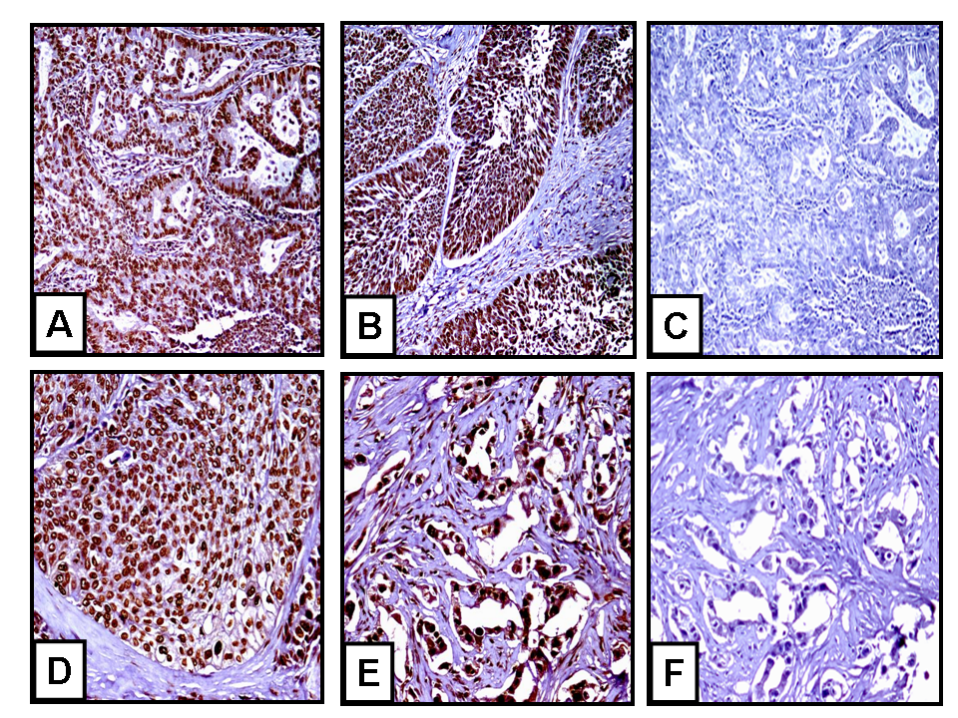
**CXCR4 expression on both tumor cells of primary tumors and metastatic lesions**. A) adenocarcinoma, B) squamous cell carcinoma, C) adenocarcinoma (negative control), D) lymph node metastasis, E) bone metastasis, and F) bone metastasis (negative control). Magnification is at 100× for A-C and 200× for D-F.

Immunostaining for CXCR4 expression on these NSCLC tissue microarrays confirmed significant expression of CXCR4 on both tumor cells of primary tumors (Figure 1A and 1B) and metastatic lesions (Figure 1D and 1E). CXCR4 is expressed on a majority (>80%) of the tumor cells in either adenocarcinoma or squamous cell carcinoma lung tumor specimens (Figure 1A and 1B, respectively). All of the squamous cell carcinoma or adenocarcinoma lung tumor specimens examined expressed positive staining for CXCR4. There was no evidence for non-specific staining with the control antibody (Figure 1C and 1F). In addition, there was little evidence of CXCR4 immunolocalization on host responding cells, such as tumor-associated macrophages, fibroblasts and endothelial cells (Figure 1). Moreover, we found that the expression of CXCR4 was a significant biomarker of metastatic NSCLC in various tissues (Figure 1D and 1E). In contrast, we found no difference in protein levels of CXCL12 in NSCLC tumors (squamous cell carcinoma and adenocarcinoma), as compared to normal lung tissue (data not shown).

### CXCR4 Expression and Pan-cytokeratin levels are Elevated in Blood of Patients with NSCLC

#### Patient Characteristics

Sixteen patients with NSCLC were enrolled to this study for the analysis of CXCR4 and pan-cytokeratin expression on circulating tumor cells. Eight females and eight males with an average age of 69 years were studied. Two patients had stage IA NSCLC, one had stage IIIA, one had stage IIIB and twelve had stage IV disease. Fourteen patients had been previously untreated and 2 subjects received 1 prior systemic treatment for metastatic disease. Only 2 patients had never smoked in this group (Table 1). The median survival for the group was 60 weeks, (range 6-not reached).

**Table 1:** Patient Characteristics

	All patients (N = 16)	Low risk group (CXCR4 ≤ 2500 cells/ml) (n = 11)	High risk group (CXCR4 > 2500 cells/ml) (n = 5)
Gender			
F	8	5	3
M	8	6	2
			
Age			
Mean ± SD	69 ± 10	67 ± 11	74 ± 6
Min-max, median	42–83, 73	42–79, 70	67–83, 73
			
Stage			
IA	2	2	0
IIIA	1	0	1
IIIB	1	1	0
IV	12	8	4
			
ECOG PS			
0	10	7	3
1	6	4	2
			
Histology			
Adenocarcinoma	6	4	2
Large cell	1	0	1
Squamous cell	2	1	1
Not Specified	7	6	1
			
Tobacco (pack-years)			
0	2	2	0
1–20	4	3	1
>20	10	6	4
			
# of prior treatments			
0	14	9	5
1	2	2	0
			
Mean Survival (weeks)	65 ± 50	79 ± 46	34 ± 50
Median Survival (weeks)	60 (6-NA)	NA (15-NA)	14 (6-NA)*

NA, not achieved

*p = 0.03 and hazard ratio of 0.23 (95% CI, 0.02–0.79) for median survival of low risk group versus high risk group (log-rank test).

#### CXCR4 and Pan-cytokeratin Expression in Peripheral Blood

**Figure 2:**
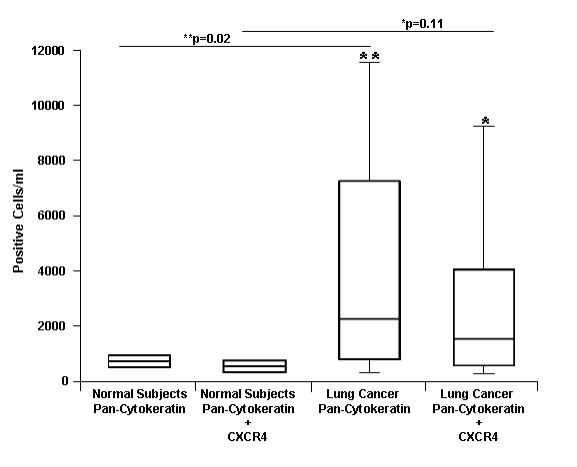
**Circulating pan-cytokeratin positive cells and CXCR4/pan-cytokeratin positive cells in 16 patients with NSCLC and 10 normal subjects**. CXCR4 and pan-cytokeratin were measured by FACS from normal healthy donors or patients with NSCLC (*p = 0.11, **p = 0.02). Horizontal line represents mean.

We hypothesized that CXCR4 and cytokeratin would be increased on circulating cells of patients with NSCLC. To determine CXCR4 expression on mononuclear cells expressing pan-cytokeratin, we measured human cytokeratin 14, 15, 16, and 19 (pan-cytokeratin) along with CXCR4 on peripheral blood mononuclear cells (PBMC) by FACS in 16 NSCLC patients and 10 normal controls. CXCR4 and pan-cytokeratin expression was elevated on circulating cells of patients with NSCLC. We found pan-cytokeratin expression was significantly increased (p = 0.02) on PBMC of patients with NSCLC when compared to normal controls (Figure 2). Combined pan-cytokeratin/CXCR4 expression was increased in patients with NSCLC, but the result was not significant (p = 0.11).

#### Low Circulating CXCR4 and Pan-cytokeratin Expression Predicts for Improved Survival

**Figure 3:**
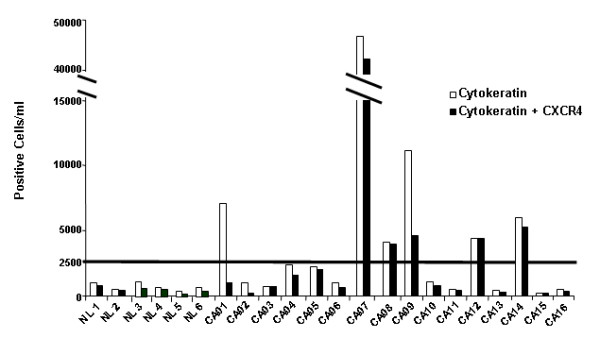
**CXCR4 expression on circulating pan-cytokeratin positive cells in patients with NSCLC**. Individual patient data for CXCR4 and pan-cytokeratin–these were measured by FACS from normal healthy donors (NL) or patients with NSCLC (CA). Results from six representative normal healthy donors are provided.

**Figure 4:**
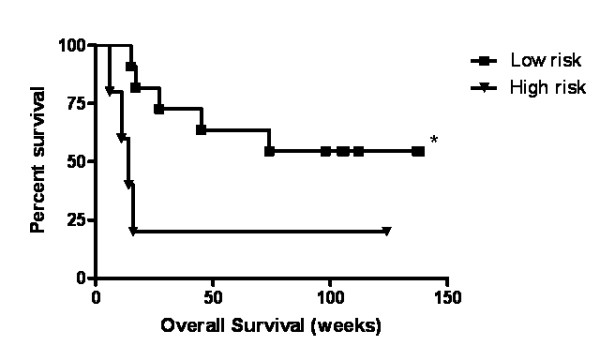
Survival by CXCR4 levels, ≤ 2500 cells/ml (low risk group, black square) vs. >2500 cells/ml (high risk group, black triangle) (*p = 0.03).

CXCR4 was elevated on circulating pan-cytokeratin positive cells in sixteen patients with NSCLC (Figure 3). In these patients, we evaluated survival based on co-expression of pan-cytokeratin and CXCR4. Using a cut point of 2500 CXCR4 positive cells/ml, we found a significant improvement in overall survival for patients with low circulating pan-cytokeratin/CXCR4-positive cells when compared to patients with high levels of circulating pan-cytokeratin/CXCR4-positive cells (p = 0.03) (Table 1). Despite the small sample size, the hazard ratio for survival in the group with low expression of pan-cytokeratin/CXCR4-positive cells was 0.23 (95% confidence interval (CI), 0.02–0.79). The median overall survival (OS) for patients with NSCLC who had low levels of CXCR4 expression on MNC (≤ 2500 CXCR4 positive cells/ml) was not reached versus a median OS of 14 weeks for those with a CXCR4 expression level > 2500 CXCR4 positive cells/ml (Figure 4).

## Discussion

The microenvironment and receptor signaling of the tumor plays a role in promoting the expression of genes that may be relevant in promoting tumor metastases. Chemokines are a group of homologous, yet functionally pleiotropic proteins that directly mediate cell migration and activation and play a role in regulating angiogenesis and metastases [25]. The CXC chemokine, CXCL12, and its receptor, CXCR4 promote metastases of a variety of solid tumors including non-small cell lung cancer (NSCLC) [9,16,20,26]. In a human NSCLC SCID mouse chimera, neoplastic cells at secondary metastatic sites significantly upregulate CXCR4 [26]. Furthermore, CXCL12, the ligand for CXCR4, has been shown to be maximally expressed in the organs of metastasis in both lung and breast cancers [9,16]. This concept supports the notion that expression of CXCR4 on tumor cells may represent a critical biomarker for their propensity to metastasize. This study was performed to evaluate the hypothesis that CXCR4 may be a marker for circulating tumor cells in metastatic NSCLC.

CXCR4 may play a role in the transition of a tumor from non-metastatic to its malignant phenotype [27]. In this study, we demonstrated that CXCR4 is expressed on primary lung tumors as well as metastatic lesions. This is consistent with previous findings that elevated levels of CXCR4 expression in NSCLC tumors was associated with clinical metastases [28]. Moreover, CXCR4 expression in squamous cell carcinoma of the head and neck could be used to identify tumor cells with increased metastatic potential [29]. Thus, identifying cells with increased CXCR4 expression may be useful to predict the metastatic potential of a tumor and provides a mechanism for inhibiting tumor progression.

We also demonstrate that circulating pan-cytokeratin and CXCR4 positive cells are more prevalent in patients with NSCLC than normal subjects. Circulating pan-cytokeratin cells were significantly elevated in NSCLC patient when compared to normal subjects. When evaluating pan-cytokeratin and CXCR4 positive together, we saw a trend toward increased levels of CXCR4 expression in NSCLC patients, but this was not statistically significant. The reasons for this observation may be due to our limited sample size since the pool of circulating CXCR4 positive cells may represent cancer stem cells[9] and comprise a smaller proportion of the cells. Similar findings have been described in metastatic renal cell carcinoma (RCC). Pan, et al. demonstrated that CXCR4 expression was markedly increased on circulating pan-cytokeratin+ cells of patients with metastatic RCC, as compared to normal control subjects, suggesting that these cells were comparable with circulating malignant cells [30]. CXCR4 was the predominant biomarker on pan-cytokeratin+ cells in the circulation of patients with metastatic RCC, suggesting that the presence of CXCR4 expression on these cells may directly correlate with metastatic RCC. Furthermore, we found that elevated co-expression of pan-cytokeratin and CXCR4 may be a prognostic marker for patients with advanced disease, as most patients in this study had stage III or IV lung cancer. Elevated co-expression of pan-cytokeratin and CXCR4 was significantly associated with poor survival in NSCLC.

CXCR4 may be important in our search to identify a biomarker or group of markers that offer the ability to detect micrometastatic disease in the blood before systemic recurrence or progression. A wide variety of biomarkers have been studied to this end, and implicated in determining prognosis for NSCLC. Thyroid transcription factor 1 (TTF1) is responsible for regulating gene transcription in the organogenesis of both thyroid and lung [31]. It has been an important marker in identifying primary lung cancers. TTF1 has been described as a potential prognostic marker associated with improved survival for patients with stage I adenocarcinoma of the lung [32]. Epithelial cellular adhesion molecule (Ep-CAM) has also been used to ascertain disseminated tumor cells in patients with surgically resectable NSCLC, and circulating levels have been associated with reduced disease-free survival [33,34]. A recent study by Zhu et al developed immunomarker-support vector machines-based prognostic classifiers for stage IB NSCLC, which were associated with survival [35]. The models integrate age, cancer cell type and either 5 or 19 markers including, *CD34MVD, p21ras, p21WAF, BCL2*, caspase-9, low-molecular-weight cytokeratin, high-molecular-weight cytokeratin, cyclooxygenase-2, *EMA, HER2*, matrix metalloproteinases (MMP)-2, MMP-9, *p16, p27kip1, p53*, vascular endothelial growth factor, β- catenin, and *tissue inhibitors of metalloproteinases (TIMP)-1 and TIMP-2*. In another study, Nagrath et al identified a highly sensitive method to evaluate circulating tumor cells from blood samples, which may correlate with tumor response to therapy [36]. Exploring these markers and techniques may prove beneficial for detecting micrometastatic disease in NSCLC. Exploiting signaling pathways involved with associated markers may be important for inhibiting micrometastatic disease to improve outcomes.

The expression of CXCR4 and HIF-1α have been linked in NSCLC [26]. Hypoxia and more specifically HIF-1α has been found to be a critical transcription factor for gene expression of CXCR4 [37-39]. Moreover, VHL can negatively regulate the expression of CXCR4, owing to its capacity to target HIF-1α for degradation under normoxic conditions [37,38]. This process may be suppressed under hypoxic conditions in cells allowing HIF-1α-dependent induction of CXCR4 expression [37,38,40]. These findings suggest that HIF-1α/VHL may play a significant role in regulating the expression of CXCR4 on tumor cells, and further understanding this molecular mechanistic link between HIF-1α and CXCR4 expression may allow the discovery of a novel means to intervene and impact on reducing metastatic NSCLC. SDF-1/CXCL12 is also regulated by HIF-1 in endothelial cells, increasing migration of circulating CXCR4-positive cells to areas of ischemic tissue. Blocking CXCL12 or CXCR4 inhibited the recruitment of these cells to sites of regenerating tissue [41]. Hypoxia, and particularly HIF-1α, has also been shown to regulate the expression of CXCR4 in cancer, including NSCLC [38,40,42,43]. Inhibition of chemokine signaling pathways with small molecule inhibitors has shown promise as a therapeutic option for regulating NSCLC growth and metastasis [26,44-46]. Further understanding of the mechanisms involved in CXCR4-mediated metastasis, and their interactions with other pathways important in NSCLC, may lead to more optimal therapeutic strategies in this disease.

## Conclusion

These findings suggest that the CXCL12/CXCR4 biological axis may be a critical determinant for the metastatic potential of NSCLC. Therefore, further understanding the molecular mechanisms that are involved in the regulation of CXCR4 expression on tumor cells could lead to potential targets to modify the expression of CXCR4 and impact on metastases. Blocking these pathways may have a significant impact on the propensity for NSCLC to invade and metastasize and therefore, the future of treatment for the disease. In addition, delineation of how chemokine ligands and receptors modulate the malignant phenotype will provide novel insights into the pathogenesis of lung carcinoma. These results are not definitive, but hypothesis-generating. In future studies, we will prospectively evaluate the prognostic significance of pan-cytokeratin/CXCR4-positive cells, and determine the mechanisms involved in the regulation of CXCR4 expression on tumor cells.

## Competing interests

The authors declare that they have no competing interests.

## Authors' contributions

KR participated in study conception, design, data acquisition, interpretation of data and drafting of the manuscript. RF participated in study design, data acquisition, and drafting of the manuscript. MB participated in data acquisition and analysis. SD participated in study conception, design and drafting of the manuscript. RE participated in data analysis. RS participated in study conception, design, interpretation of data and drafting of the manuscript. All authors read and approved the final manuscript.

## Pre-publication history

The pre-publication history for this paper can be accessed here:

http://www.biomedcentral.com/1471-2407/9/213/prepub
